# Novel Strategies for Cancer Combat: Drug Combination Using Repurposed Drugs Induces Synergistic Growth Inhibition of MCF-7 Breast and HT-29 Colon Cancer Cells

**DOI:** 10.3390/cimb44100335

**Published:** 2022-10-16

**Authors:** Diana Duarte, Inês Guerreiro, Nuno Vale

**Affiliations:** 1 OncoPharma Research Group, Center for Health Technology and Services Research (CINTESIS), Rua Doutor Plácido da Costa, 4200-450 Porto, Portugal; 2 Faculty of Pharmacy, University of Porto, Rua Jorge Viterbo Ferreira, 228, 4050-313 Porto, Portugal; 3 CINTESIS@RISE, Faculty of Medicine, University of Porto, Alameda Professor Hernâni Monteiro, 4200-319 Porto, Portugal; 4 Department of Community Medicine, Health Information and Decision (MEDCIDS), Faculty of Medicine, University of Porto, Rua Doutor Plácido da Costa, 4200-450 Porto, Portugal

**Keywords:** drug repurposing, drug combination, synergism evaluation, CNS drugs, antimalarial drugs, cancer therapy

## Abstract

Our group developed a new model of drug combination consisting of the use of antineoplastic drugs and different repurposed drugs, having demonstrated that antimalarial and central nervous system (CNS) drugs have a promising anticancer profile as standalone agents, as well as in combined regimens. Here, we evaluated the anticancer profiles of two different CNS drugs (edaravone and quetiapine), both alone and in combination with antineoplastic agents for breast and colon cancer, to explore whether these repurposed drugs could synergistically enhance the anticancer potential of chemotherapeutic drugs. We also developed a new model of combination using two repurposed drugs, to explore whether this model of combination could also be suitable for application in breast and colon cancer therapy. MCF-7 and HT-29 cancer cells were incubated for 48 h with each individual drug (0.01–100 µM) to determine their IC_50_. Cells were then treated with the IC_50_ value for doxorubicin or paclitaxel (MCF-7) or 5-fluorouracil (HT-29) and combined with increasing concentrations of edaravone or quetiapine for 48 h. Both cell lines were also treated with a combination of two antimalarial drugs (mefloquine and pyronaridine) or two CNS drugs (fluphenazine and sertraline) for 48 h. We found that the use of quetiapine in combined therapies seems to synergistically enhance the anticancer activity of doxorubicin for the management of breast cancer. Both CNS drugs significantly improved the cytotoxic potential of 5-fluorouracil in HT-29 cells, with quetiapine synergistically interacting with the antineoplastic drug in this drug combination. Regarding the combination of repurposed drugs, only found one synergic combination regimen (sertraline IC_50_ plus variable concentrations of fluphenazine) with anticancer potential against HT-29 colon cancer cells was found. Taken together, these results suggest that quetiapine and edaravone can be used as adjuvant agents in chemotherapy for colon cancer. It was also found that the combination of repurposed drugs, specifically the CNS drugs sertraline and fluphenazine, may have an interesting profile for application in colon cancer novel therapies.

## 1. Introduction

The latest cancer statistics provided by the American Cancer Society estimate that, in 2022, more than 1,900,000 new cancer cases will be diagnosed and there will be about 600,000 cancer deaths, in the United States alone [1]. It is also estimated that breast and colorectal cancers will occupy the second and third positions as the most diagnosed types of cancer, with more than 40,000 and 50,000 estimated deaths, respectively [1]. Antineoplastic drugs commonly used in chemotherapy are not fully efficient, with some lack of selectivity and high toxicity, which results in severe side effects.

5-fluorouracil (5-FU) is an antineoplastic drug commonly used for the management of colon cancer, although its use is limited by its short half-life, high cytotoxicity, and low bioavailability [2]. One of the most used antineoplastic drugs in breast cancer therapy is paclitaxel (PTX), a mitotic inhibitor that causes mitotic arrest and cell apoptosis. However, several studies reported that different cancer cells develop resistance to this drug and that the use of this drug induces the appearance of undesired side effects, limiting the use of this chemotherapeutic agent [3]. Another drug used for the treatment of breast cancer is doxorubicin (DOX), an antineoplastic drug that intercalates with the DNA chain leading to cell cycle arrest and apoptosis [4]. Nevertheless, the use of this drug is also limited by its cardiotoxicity and side effects. Moreover, the use of chemotherapeutics, particularly DOX, is associated with gonad toxicity, which leads to the induction of oxidative stress and consequently impairs ovarian function [5]. Indeed, this was demonstrated in a recent study [6] where it was found that a DOX intraperitoneal injection in ICR mice causes an increase in nuclear factor erythroid 2-related factor 2 (Nrf2) expression, haem oxygenase (HO-1) and catalase, as well as a reduction in glutathione peroxidase and superoxide dismutase 1 (SOD-1) expressions [6]. Under normal conditions, the transcription factor Nrf2 plays an important role in the protection of cells against oxidative stress [7]. The increase in ROS leads to the activation of the Nrf2 pathway, which induces the expression of antioxidant enzymes, such as HO-1, catalase, glutathione peroxidase, and SOD-1 that protect cells against oxidative damage [7]. Nevertheless, in cancer cells, Nrf2 plays an important role in the development of drug resistance, impairing drug-mediated oxidative stress and contributing to cancer cell survival, by inhibiting apoptosis and promoting cell proliferation [7]. Furthermore, it was found that DOX significantly decreases the number of primordial, primary, preantral, and antral follicles, while it increases the number of atretic follicles [6]. These findings must be considered by clinicians, especially when proposing DOX chemotherapy treatments to young women who would like to have children. Consequently, the development of novel oncologic therapeutic strategies, with safer and more effective profiles, is imperative to help improve the outcomes of the treatment and the quality of life of cancer patients.

Several signs of progress were demonstrated in the research into novel therapeutic strategies for cancer, with drug combination and drug repurposing strategies gaining attention by the scientific community in recent years. Drug repurposing, also known as drug repositioning, is an alternative to de novo drug development and can accelerate access to novel therapeutic agents for cancer patients [8]. It refers to the strategy of finding new therapeutic indications for drugs that are already approved by the Food and Drug Administration (FDA) for clinical use [9]. This strategy is advantageous compared with the development of new drugs, as such drugs already have well-defined toxicological and pharmacokinetic profiles [10], resulting in a more economic and faster translational process, usually increasing the chances of the drug progressing to clinical trials [11]. Moreover, most repurposed drugs may be taken orally with good tolerance, and are generic medicines, making them more affordable than newly patented drugs [10]. Nevertheless, it is important to consider the novel side effects that may not be described for its original indication and that can appear upon a new therapeutic indication [9]. For this reason, the drug repurposing process still requires clinical trials to further validate its novel application [9]. Drug repurposing was already applied for different indications, with pre-clinical and clinical safety data available. The most well-known examples are the use of sildenafil for erectile dysfunction and pulmonary hypertension, and thalidomide for leprosy and multiple myeloma [12]. Antibiotics, antidepressants, antipsychotic drugs, cardiovascular drugs, microbiological agents, and non-steroidal anti-inflammatory drugs (NSAIDs) are among the repurposed drugs already proposed for cancer therapy [13].

Drug combination refers to the use of cocktails with two or more drugs, usually with different mechanisms of action and drug targets [14], that can contribute to targeting different oncologic pathways at the same time [15,16,17,18], increasing the efficacy of the therapy and decreasing the chances of the development of drug resistance [19]. The use of drugs in combination is particularly advantageous when each of the drugs acts on a different target or signaling pathway, thereby producing a synergistic effect, which helps reduce the required drug concentrations for each individual drug [20]. Therefore, the use of drug combinations can help increase the success rate of drug repurposing screens [20]. The combination of repurposed drugs with antineoplastic agents already demonstrated interesting results, especially when the usual anticancer monotherapy failed in the safety and tolerability of oncological patients [21]. Our group already used these two therapeutic strategies for the development of novel strategies for breast and colon cancer treatment. In our previous work, we developed a new combination model consisting of the use of antineoplastic drugs and several repurposed drugs from different classes [22,23,24]. These studies aimed to find repurposed drugs with promising anticancer profiles against two different cell lines: MCF-7 (breast cancer) and HT-29 (colon cancer) and, at the same time, to evaluate whether they could synergistically improve the anticancer activity of antineoplastic drugs commonly used for these types of cancer. To do so, we evaluated the cytotoxic effects of several antimalarial and central nervous system (CNS) drugs in these cell lines and combined them with chemotherapeutic agents (PTX and DOX for breast cancer, and 5-FU for colon cancer). We successfully demonstrated these repurposed drugs can be used as standalone agents for breast and colon cancer therapy and that their use in combination regimens is a promising strategy to improve the efficacy of antineoplastic agents [22,23,24].

In this work, we first decided to evaluate whether the CNS drugs edaravone and quetiapine would also have a promising anticancer profile for drug repurposing in breast and colon cancer therapy, and whether they could improve the anticancer activity of DOX and PTX in MCF-7 breast cancer cells, and 5-FU in HT-29 colon cancer cells. Edaravone and quetiapine were included in this study based on our previous findings, which demonstrated that several CNS drugs presented anticancer effects. Nevertheless, no reports were found on the potential of edaravone and quetiapine as chemotherapeutic agents. In the second part of the work, we develop a new model of combination consisting of the combined use of two repurposed drugs. To do so, and based on our previous studies, we selected the four repurposed drugs with the most promising anticancer profiles (based on their IC_50_ value). Collectively, we studied the combined use of two antimalarial (pyronaridine and mefloquine) and two CNS drugs (fluoxetine and sertraline) on the MCF-7 and HT-29 cancer cell lines. Drug interactions in all combined pairs were evaluated by the quantification of the Combination Index, which indicates whether the combinations are synergic, additive, or antagonist. Synergism is a desirable aspect when designing drug combinations as this means that lower doses of each drug can be used to achieve a determined cytotoxic effect.

We found that edaravone and quetiapine do not have an adequate anticancer profile to be used as single agents in either MCF-7 or HT-29 cancer cells. Nevertheless, the use of quetiapine in combined therapies seems to enhance the anticancer activity of DOX (but not PTX) for the management of breast cancer. In the combination of DOX and quetiapine, it was also demonstrated that the two drugs interact synergistically. In the case of colon cancer, both drugs significantly improved the cytotoxic potential of 5-FU, with quetiapine synergistically interacting with the antineoplastic drug in this drug combination. Regarding the combination of repurposed drugs, this model of combination only presented significant results when using CNS drugs, specifically when combining sertraline IC_50_ plus variable concentrations of fluphenazine, only in the HT-29 colon cancer cells.

## 2. Materials and Methods

### 2.1. Reagents

Cell culture reagents (cell culture medium, phosphate-buffered saline (PBS), fetal bovine serum (FBS), and penicillin–streptomycin (pen–strep) solution) were purchased from Millipore Sigma (Merck KGaA, Darmstadt, Germany). Other cell culture reagents were bought from Gibco (Thermo Fisher Scientific, Inc, Waltham, MA, USA). Fluphenazine (cat. no. F4765), pyronaridine (cat. no. P0049), edaravone (cat. no. M70800), 5-FU (cat. no. F6627), and Thiazolyl Blue Tetrazolium Bromide (MTT, cat. no. M5655) were obtained from Sigma-Aldrich (Merck KGaA, Darmstadt, Germany). Sertraline (cat. no. 14839), quetiapine (cat. no. 14152), and DOX (cat. no. 15007) were acquired from Cayman Chemical (Ann Arbor, MI, USA). PTX (cat. no. 1097) was obtained from Tocris Bioscience (Bristol, UK). Mefloquine (cat. no. sc-211784) was purchased from Santa Cruz Biotechnology (Dallas, TX, USA).

### 2.2. Cell Culture

Cytotoxicity of each treatment was performed using two cell lines: MCF-7 breast and HT-29 colon cancer (ATCC, American Type Culture Collection, Manassas, VA, USA). The MCF-7 and HT-29 cells were kept in DMEM and McCoy’s cell culture medium, respectively, supplemented with 10% FBS and 100 U/mL penicillin plus 100 μg/mL streptomycin. The cells were maintained at 37 °C in an incubator with 95% air and 5% CO_2_. Both cell lines were trypsinized using a 0.25% trypsin-EDTA solution when at 70–80% confluence.

### 2.3. Drug Treatment

On the day before cell treatment, 200 µL of MCF-7 and HT-29 cell suspension was seeded in 96-well plates at a density of 10,000 cells/well. Cells were allowed to grow and adhere for 24 h and then each well was aspirated and 200 µL of drug-containing media was added. All drugs were prepared in 100% DMSO and diluted to 0.1% DMSO using culture media. Control cells were treated with vehicle (0.1% DMSO). No significant changes were seen in control cells with and without vehicle. After 48 h treatment, morphological analysis was performed, and then cell viability was evaluated by an MTT assay.

Prior to combination treatments, cells were incubated with each drug alone to determine their IC_50_. To do so, both cell lines were incubated with increasing concentrations (0.01–100 µM) of a single drug for 48 h. The IC_50_ value was calculated as the concentration causing 50% cell growth inhibition compared with control cells.

For the combined treatments with antineoplastic drugs and CNS drugs, the MCF-7 cells were treated with the IC_50_ value for DOX (0.17 µM) or PTX (0.44 nM) and combined with increasing concentrations (0.01–100 µM) of edaravone or quetiapine for 48 h. In the case of the HT-29 cells, the treatment consisted of the combination of 5-FU at IC_50_ (3 µM) with increasing concentrations (0.01–100 µM) of edaravone or quetiapine for 48 h.

For the combined treatments with the repurposed drugs, both cell lines were treated with a combination of two antimalarial drugs (mefloquine and pyronaridine) or two CNS drugs (fluphenazine and sertraline) for 48 h. Regarding the combination of antimalarial drugs, cells were incubated with mefloquine IC_50_ plus increasing concentrations (0.01–100 µM) of pyronaridine and vice-versa. The same experimental protocol was followed for the combination of CNS drugs.

### 2.4. Microscopic Observation

After each treatment and before performing the MTT assays, cells were examined using a Leica DMI 6000B microscope coupled to a Leica DFC350 FX camera, and images were analyzed using Leica LAS X imaging software (v3.7.4, Leica Microsystems, Wetzlar, Germany).

### 2.5. Viability Assay

Cell viability was assessed using an MTT assay. Briefly, after drug treatment, the cell media was removed carefully and 100 μL of MTT solution (0.5 mg/mL in PBS) was added to each well. Cells were then incubated with this reagent for 3 h in an incubator at 37 °C protected from light. After this period, the MTT solution was removed carefully from each well and replaced by 100 μL/well of DMSO, to help dissolve the formazan crystals. Absorbance was measured using an automated microplate reader (Tecan Infinite M200, Tecan Group Ltd., Männedorf, Switzerland) at a wavelength of 570 nm.

### 2.6. Data Analysis

The concentration–response curves were obtained using nonlinear regression in GraphPad Prism 8 software (GraphPad Software Inc., San Diego, CA, USA). To do so, we first normalized the cell viability of the treated cells to the cell viability of the control cells. The normalized cell viability fractions were then plotted against the logarithm of drug concentrations and IC_50_ was calculated.

### 2.7. Synergism Evaluation

The synergism evaluation was performed using the MTT cell viability results obtained after single and combined treatments in both cell lines. Synergism was assessed by the calculation of the CI using CompuSyn Software (ComboSyn, Inc., New York, NY, USA). This method is based on the unified theory proposed by Chou and Talalay [25] and assumes that all drugs act through entirely different mechanisms [26]. The graphical representation consisted in plotting the CI values on the *y*-axis against the Fa on the *x*-axis; this indicates the pharmacological interactions of two drugs in combination, representing synergism, additivity, or antagonism when its value is under, equal, or above 1, respectively.

### 2.8. Statistical Analysis

The results obtained by the MTT assays were statistically analyzed using GraphPad Prism 8 software (GraphPad Software Inc., San Diego, CA, USA), after three independent experiments. Results are expressed as mean ± SEM and statistical differences between treatment groups and control cells were evaluated using the Student’s *t*-test and one-way ANOVA test. Results were considered statistically significant at *p* < 0.05.

## 3. Results

### 3.1. Combination of Antineoplastic Drugs with Quetiapine and Edaravone for Breast and Colon Cancer Therapy

#### 3.1.1. MCF-7 Results

First, we analyzed the antitumor potential of the CNS drugs edaravone and quetiapine in the MCF-7 breast cancer cell line, to confirm their efficacy in this type of cancer. Cells were treated with increasing concentrations (0.01–100 μM) of each drug for 48 h and cell viability was measured using an MTT assay. The results of the MTT assays for edaravone and quetiapine are represented in Supplementary Figure S1. The MTT results regarding edaravone demonstrate this drug lacks anticancer activity against this cancer cell line (Figure S1A), as further reflected in the dose–response curve of edaravone in Figure S1B. The results for the MCF-7 cells treated with quetiapine revealed significant anticancer activity by this drug at the highest concentration (100 µM), with a reduction of about 50% in cell viability; nevertheless, little cytotoxic effect was observed at concentrations under 50 µM (Figure S1C). The dose–response curve resulted in an IC_50_ value of more than 50 µM. These results demonstrate these CNS drugs are not adequate as standalone agents for breast cancer therapy and justify their investigation as adjuvant agents in combination therapies.

We next evaluated the combination of two common antineoplastic drugs used in breast cancer therapy (PTX and DOX) with edaravone and quetiapine, to determine whether these repurposed drugs could effectively enhance the anticancer activity of the chemotherapeutic drugs. To do so, we combined increasing concentrations (0.01–100 μM) of edaravone and quetiapine with the IC_50_ values of PTX and DOX. These values were already obtained in our previous work [23]. Cell viability was assessed by an MTT assay and morphological evaluation was also performed. The results regarding the combination of DOX plus edaravone are shown in Figure S2. Analyzing the morphological results (Figure S2A) and MTT results (Figure S2B), it is possible to observe that the combined effects of edaravone and DOX are very similar to the effect of DOX alone, for all concentrations, demonstrating that the cytotoxic effect seen for these combinations may be a result of the strong anticancer activity of DOX alone. The same conclusions can be applied to the combination of edaravone with PTX (Figure S3).

**Figure 1:**
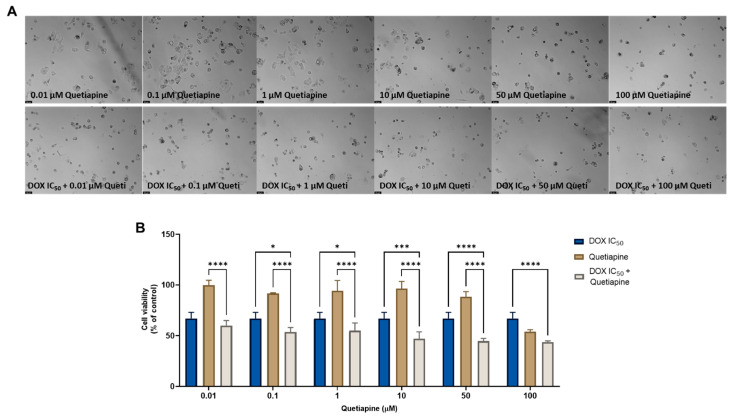
Morphological analysis (**A**) and cellular viability (**B**) results of MCF-7 cells after a single and combined treatment with DOX and quetiapine. Cells were incubated with increasing concentrations of quetiapine, alone and combined with a fixed concentration (IC_50_) of DOX for 48 h. Cell viability was evaluated using an MTT assay. Experiments were performed three times independently (*n* = 3). *, *** and **** indicate significative results at *p* < 0.05, *p* < 0.001 and *p* < 0.0001, respectively.

On the other hand, the combination of quetiapine and DOX revealed significant morphological changes in the cell structure and cell number of the MCF-7 cells (Figure 1A). These observations are compatible with the results obtained by the MTT assay (Figure 1B), where a significative reduction was found in the cell viability of the MCF-7 cells treated with the combination of DOX plus 0.1, 1, 10, and 50 µM of quetiapine compared with both drugs alone, demonstrating the promising anticancer profile of this drug combination against this cell line.

Regarding the combination of PTX + quetiapine, it was found this drug combination does not have significant anticancer effects compared to PTX alone, either in the morphological analysis (Figure S4A) or in the MTT assay (Figure S4B), demonstrating only a significant reduction in cell viability in comparison with quetiapine alone. This demonstrates the anticancer effect of the combination is mainly attributed to the anticancer activity of the antineoplastic drug.

**Figure 2:**
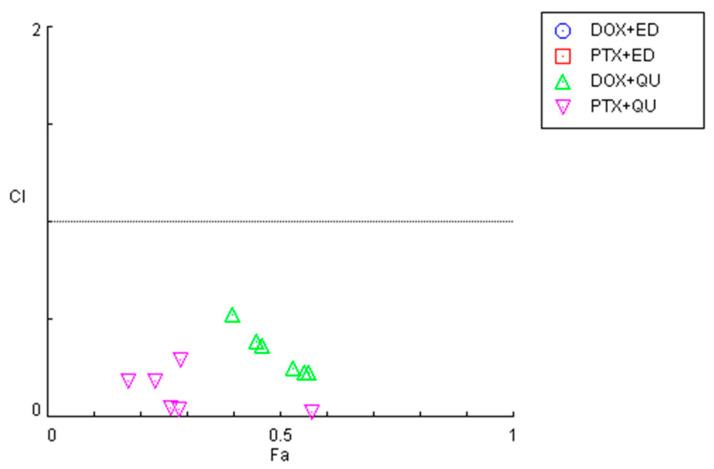
Graphical representation of combination index plot obtained from the CompuSyn Report for the combinations of DOX + edaravone (blue), PTX + edaravone (red), DOX + quetiapine (green) and PTX + quetiapine (pink).

To investigate the synergistic effects of the combinations of antineoplastic drugs DOX and PTX with CNS drugs quetiapine and edaravone, the combination index (CI) was calculated using the Chou–Talalay method implemented in CompuSyn software. These results are summarized in Figure 2 and Table 1. For the graphical representation, CI was plotted on the *y*-axis as a function of the effect level (Fa) on the *x*-axis. The Fa is a value between 0 and 1, meaning no effect of the drug combination on the cell viability (if Fa = 0) or full effect of the combination on reducing cell viability (if Fa = 1). CI values under 1 indicate the drugs interact synergistically, while CI > 1 means an antagonistic interaction. CI = 1 indicates that the effect of the drugs within a combination is additive.

In line with the previous MTT results, it was found that the combinations of both chemotherapeutic drugs with edaravone resulted in all pairs being antagonists (CI > 10), with Fa values being very low (all under 0.5). As the *y*-axis in Figure 2 is under 2, these points are not displayed in the graph. On the other hand, the combination of DOX and PTX with quetiapine demonstrated promising results, with all pairs being synergistic. Nevertheless, the Fa values for these combinations are under 0.6, even when using the highest concentration of quetiapine, demonstrating that, although the drugs act synergistically, the combinations themselves cannot reduce cell viability by more than 60%.

#### 3.1.2. HT-29 Results

**Figure 3:**
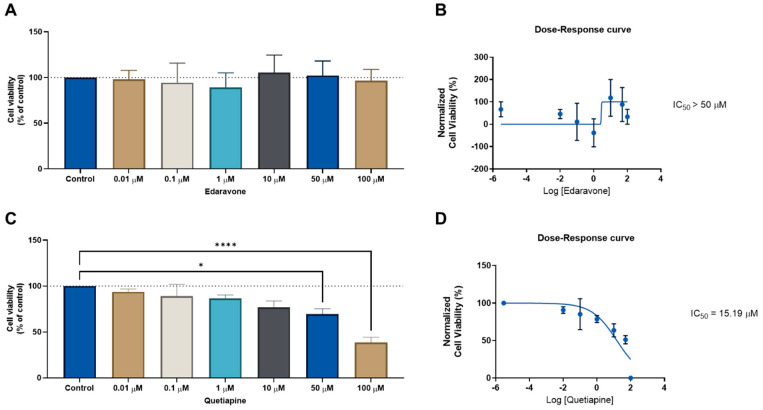
Cellular viability results for HT-29 cells after treatment with the CNS drugs edaravone (**top**) and quetiapine (**bottom**). (**A**) MTT results and (**B**) dose–response curve for HT-29 cells treated with edaravone. (**C**) MTT results and (**D**) dose–response curve for HT-29 cells treated with quetiapine. Cells were incubated with increasing concentrations of each drug for 48 h and cell viability was evaluated using an MTT assay. Dose–response curves were obtained by normalization of cell viability results against the logarithm of the concentration of each drug. The IC_50_ value was determined using GraphPad. Experiments were performed three times independently (*n* = 3). * and **** indicate significant results at *p* < 0.05 and *p* < 0.0001, respectively.

We next evaluated the same drug combinations in another cell line (HT-29 colon cancer) to determine whether these drugs could have anticancer effects in another type of cancer. We followed the same methodologies as previously outlined for the MCF-7 cells. First, the CNS drugs edaravone and quetiapine were evaluated alone in increasing concentrations for 48 h, to determine their ability to reduce the cell viability in HT-29 cells. This was determined by an MTT assay. The IC_50_ for each drug was then calculated using Graphpad software. In line with the MCF-7 results, the CNS drug edaravone did not demonstrate the ability to significantly reduce the viability of the HT-29 cell line in all concentrations (Figure 3A), resulting in an IC_50_ value above 50 µM (Figure 3B). Regarding the CNS drug quetiapine, it was found that this drug has a concentration-dependent anticancer effect and is able to significantly reduce cell viability in HT-29 cells at concentrations of 50 and 100 µM (Figure 3C), with an IC_50_ value of 15.19 µM (Figure 3D). These results demonstrate this repurposed drug has a higher anticancer efficacy against HT-29 colon cancer cells than MCF-7 breast cancer cells.

Next, edaravone and quetiapine were combined in increasing concentrations with the IC_50_ value for 5-FU. This drug is an antineoplastic agent commonly used for the management of colon cancer, and this combination aimed to determine whether these repurposed drugs could enhance the anticancer effect of 5-FU.

**Figure 4:**
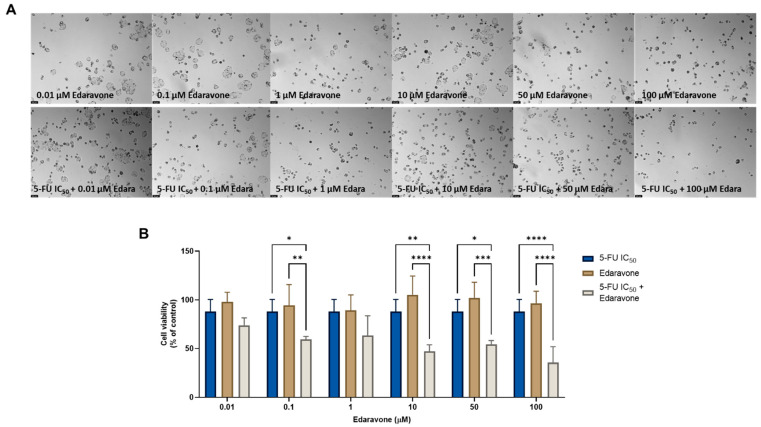
Morphological analysis (**A**) and cellular viability (**B**) results for HT-29 cells after a single and combined treatment with 5-FU and edaravone. Cells were incubated with increasing concentrations of edaravone, alone and combined with a fixed concentration (IC_50_) of 5-FU for 48 h. Cell viability was evaluated using an MTT assay. Experiments were performed three times independently (*n* = 3). *, **, *** and **** indicate significant results at *p* < 0.05, *p* < 0.01, *p* < 0.001 and *p* < 0.0001, respectively.

Regarding the combination of edaravone and 5-FU, significant anticancer effects were found compared with each drug alone, when 0.1, 10, 50, and 100 µM of edaravone were combined with 5-FU, as seen by morphological analysis (Figure 4A) and by MTT assay (Figure 4B). These results differ from those previously obtained for the MCF-7 cells, where it was found that the combination of edaravone and chemotherapeutic agents did not produce any significant effects on cell viability.

**Figure 5:**
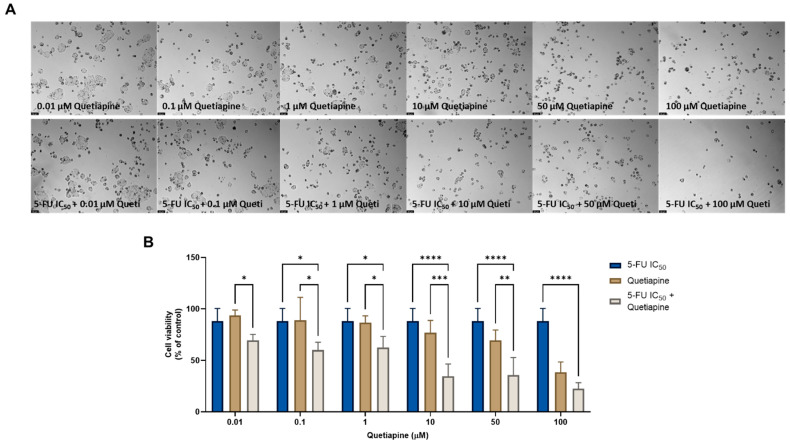
Morphological analysis (**A**) and cellular viability (**B**) results of HT-29 cells after a single and combined treatment with 5-FU and quetiapine. Cells were incubated with increasing concentrations of quetiapine, alone and combined with a fixed concentration (IC_50_) of 5-FU for 48 h. Cell viability was evaluated using an MTT assay. Experiments were performed three times independently (*n* = 3). *, **, *** and **** indicate significant results at *p* < 0.05, *p* < 0.01, *p* < 0.001 and *p* < 0.0001, respectively.

**Figure 6:**
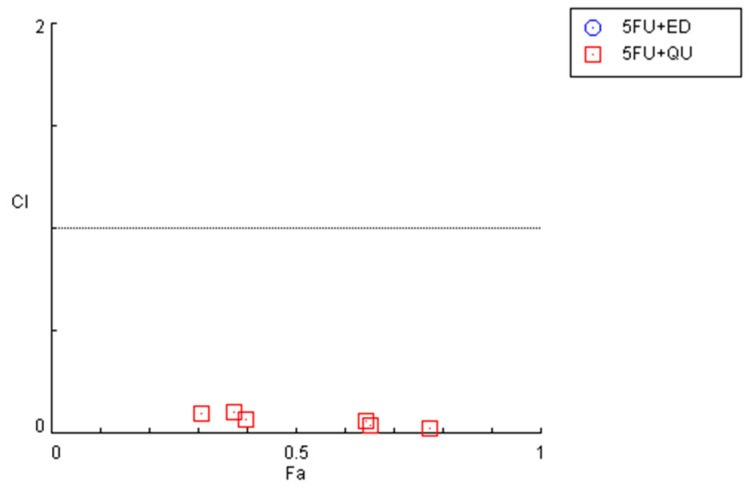
Graphical representation of combination index plot obtained from the CompuSyn Report for the combinations of 5-FU + edaravone (blue) and 5-FU + quetiapine (red).

The combination of 5-FU and quetiapine also resulted in significant anticancer effects compared with each drug alone, visible both in the analysis of cell morphology (Figure 5A) and in the MTT results (Figure 5B), for the combination of intermediate concentrations of quetiapine (0.1, 1, 10 and 50 µM). These results demonstrate that these repurposed drugs are promising adjuvant agents for combination therapies in colon cancer therapy. These promising results are also supported by the evaluation of drug synergism between 5-FU and quetiapine (Figure 6 and Table 2), where it was found that all drug pairs present a CI value under 1, indicative of synergistic interactions. Additionally, for the combination of the highest concentration of quetiapine with the IC_50_ value for 5-FU, it was found the combined effect induced about 80% of cell death in this cell line. On the other hand, the CI values obtained for the combination of 5-FU and edaravone demonstrated all drug pairs to have values above 10, indicating antagonistic effects between the drugs (Figure 6 and Table 2). Taken together, the results obtained in the two cell lines indicate that quetiapine is a more promising repurposed drug for cancer therapy, demonstrating anticancer activity as a standalone agent against HT-29 cells and as an adjuvant agent in combination regimens with DOX and 5-FU against MCF-7 and HT-29 cells, respectively.

### 3.2. Combination of Repurposed Drugs for Breast and Colon Cancer Therapy

In the second part of this study, we aimed to develop a new model of drug combination consisting of two repurposed drugs. We selected repurposed drugs with the most promising anticancer profile, taking into account our previous results [22,23,24] and those previously determined in Section 2.1 (Table 3). The drugs were selected based on their IC_50_, assuming that the lower this value, the more their anticancer potential. By analyzing Table 3, we selected the two CNS and the two antimalarial drugs with the lowest IC_50_ values in both cell lines, to confirm the viability of this model of combination for breast and colon cancer therapy. This model of combination consisted in fixing the dose of one drug and varying the dose of the other drug. To do so, we treated MCF-7 and HT-29 cells with the combinations of fluphenazine plus sertraline (and vice-versa), and with the combination of mefloquine plus pyronaridine (and vice-versa) for 48 h and evaluated cell morphology and viability after each treatment.

#### 3.2.1. MCF-7 Results

First, we evaluated the combination of mefloquine (IC_50_) with increasing concentrations of pyronaridine on the cell morphology (Figure S5A) and viability (Figure S5B) of MCF-7 breast cancer cells. The results demonstrate that this combination is not advantageous over mefloquine at all concentrations of pyronaridine, with mefloquine alone causing more than an 80% reduction in cell viability in its IC_50_ value.

We next inverted the combination and fixed the dose of pyronaridine, and varied the concentration of mefloquine. The results of the morphological analysis (Figure S6A) and for cellular viability (Figure S6B) demonstrate that the combination of 0.01, 0.1, and 1 of mefloquine with the IC_50_ for pyronaridine significantly reduces MCF-7 cell viability only when compared with mefloquine alone in these concentrations. No statistically significant combined pairs were found at any concentration compared to both drugs alone, which demonstrates that this combination is not advantageous over single drugs.

**Figure 7:**
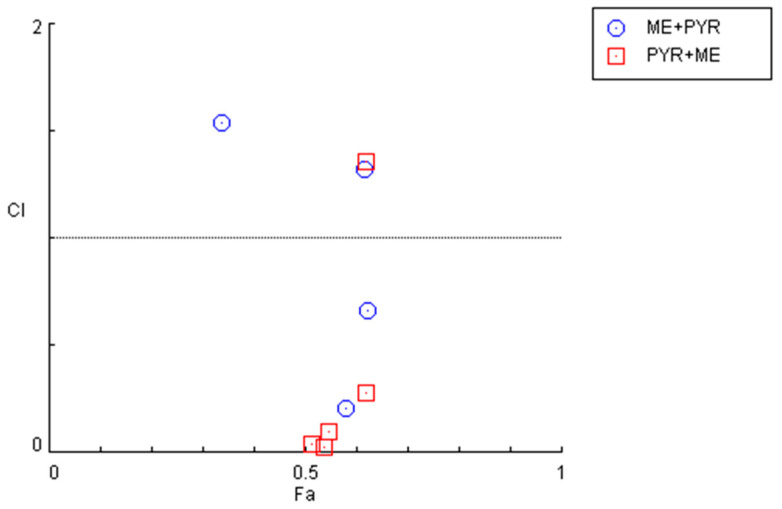
Graphical representation of combination index plot obtained from the CompuSyn Report for the combinations of mefloquine IC_50_ + variable pyronaridine (blue) and pyronaridine IC_50_ + variable mefloquine (red).

Nevertheless, an examination of the combination index for the previously described combinations (Figure 7 and Table 4) demonstrates that some pairs interact synergistically, especially when the pyronaridine concentration is fixed and the mefloquine concentration is variable. Regarding the combination of the mefloquine IC_50_ plus variable pyronaridine, CI values of 0.205 and 0.664 were found for the combination with 10 and 50 µM of pyronaridine, respectively. The combination of pyronaridine IC_50_ plus variable mefloquine demonstrated synergism for lower concentrations of mefloquine (<10 µM).

The same combination model previously described was then performed using the CNS drugs fluphenazine and sertraline. The results regarding the combination of sertraline IC_50_ and fluphenazine (0.01–100 µM) are shown in Figure S7. The results of the cell morphological analysis (Figure S7A) and MTT assay (Figure S7B) demonstrate this combination induces significant toxicity compared with sertraline alone, suggesting that the combined effect must be a result of the cytotoxicity of fluphenazine. The same conclusions can be applied to the inverse combination of fluphenazine IC_50_ and variable concentrations of sertraline (Figure S8), where it was found that the effect observed after the drug combination treatment is practically equal to sertraline alone.

**Figure 8:**
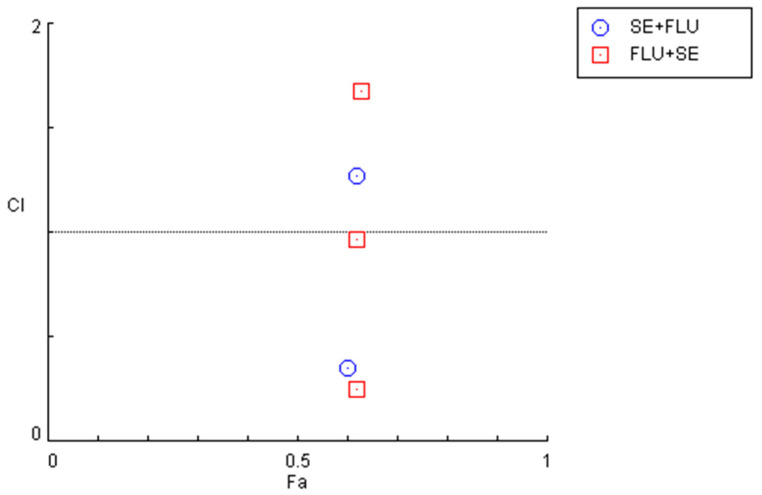
Graphical representation of combination index plot obtained from the CompuSyn Report for the combinations of sertraline IC_50_ + variable fluphenazine (blue) and fluphenazine IC_50_ + variable sertraline (red).

Indeed, after calculation of the CI values and the respective fractional effects of the drug combinations using CNS drugs in MCF-7 cells (Figure 8 and Table 5), only one synergistic pair was found for the combination of sertraline IC_50_ plus 10 µM of fluphenazine and two pairs regarding the combination of fluphenazine IC_50_ and variable doses of sertraline (10 and 50 µM).

#### 3.2.2. HT-29 Results

We next examined the combinations of repurposed drugs in another cell line (HT-29) to evaluate whether these drug pairs would have anticancer potential in another type of cancer. The effect of the combination of mefloquine (IC_50_) with increasing concentrations of pyronaridine on the cell morphology (Figure S9A) and viability (Figure S9B) of the HT-29 colon cancer cells indicates that this combination is only statistically significant when compared with the cytotoxic effect of pyronaridine alone at lower concentrations (0.01, 0.1, 1 and 10 µM). It was also found that the drug combination is not advantageous over mefloquine alone, with mefloquine alone causing more than an 80% reduction in cell viability in its IC_50_ value.

On the other hand, when analyzing the results of the reverse combination of a fixed concentration of pyronaridine (IC_50_) and variable concentrations of mefloquine, both those obtained after microscopic visualization (Figure S10A) and the viability assay (Figure S10B), it was possible to observe that the combination regimen only induces significant reductions in cell viability compared with mefloquine, and at lower concentrations (0.01, 0.1 and 1 µM). Also, at the same range of concentrations, it is possible to conclude that the anticancer activity may be a result of the cytotoxic potential of pyronaridine, while at higher concentrations (>10 µM), the combined effect may be derived from the anticancer activity of mefloquine alone.

**Figure 9:**
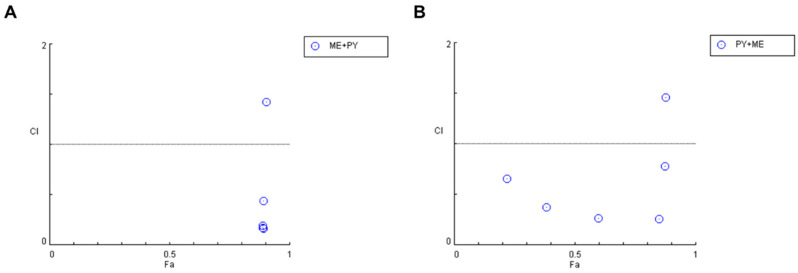
Graphical representation of combination index plot obtained from the CompuSyn Report for the combinations of (**A**) mefloquine IC_50_ + variable pyronaridine and (**B**) pyronaridine IC_50_ + variable mefloquine.

The evaluation of drug interactions shown in Figure 9 and Table 6 indicates that the combination of mefloquine IC_50_ plus pyronaridine acts synergistically when lower concentrations (< 10 µM) of pyronaridine are used. On the other hand, when combining pyronaridine at a fixed concentration plus variable concentrations of mefloquine, CI values under 1 were found for all concentrations, except for 100 µM of mefloquine, indicating synergistic interactions between these repurposed drugs.

**Figure 10:**
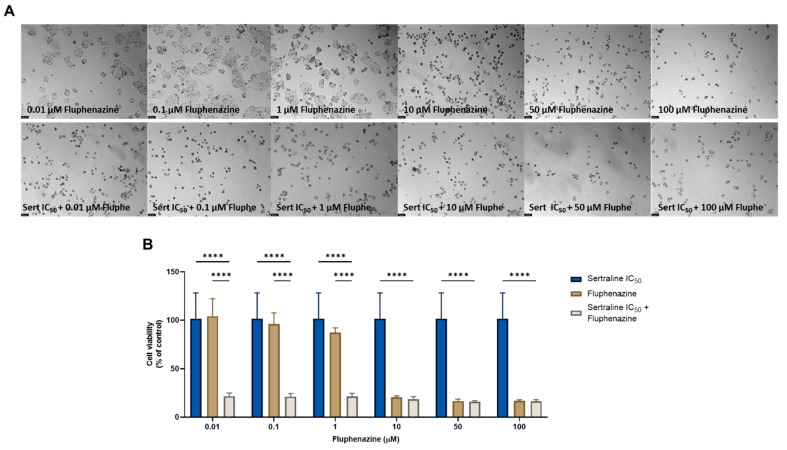
Morphological analysis (**A**) and cellular viability (**B**) results for HT-29 cells after a single and combined treatment with fluphenazine and sertraline. Cells were incubated with increasing concentrations of fluphenazine, alone and combined with a fixed concentration (IC_50_) of sertraline for 48 h. Cell viability was evaluated using an MTT assay. Experiments were performed three times independently (*n* = 3). **** indicates significant results at *p* < 0.0001.

Finally, we combined the two most potent CNS drugs in HT-29 colon cancer cells and evaluated the changes in cell morphology and cell viability after a treatment of 48 h. First, we evaluated the combination of sertraline at a fixed concentration of IC_50_ with increasing concentrations (0.01–100 µM) of fluphenazine. Analyzing both the microscopic images (Figure 10A) and the results obtained in the MTT assay (Figure 10B), it is possible to verify significant changes in the number and phenotype of the HT-29 cells treated with this combination, specifically when compared with a single treatment with fluphenazine in lower concentrations (<1 µM). Indeed, the analysis of the MTT results demonstrates a significant reduction in the viability of this cell line at treatments with the combination of sertraline and 0.01, 0.1, and 1 µM fluphenazine, in comparison with both drugs alone, demonstrating the anticancer potential of these repurposed drugs to be used in combination regimens.

On the other hand, when using the reverse combination, i.e., combining a fixed concentration of fluphenazine with increasing concentrations of sertraline, it was found that the combination was not able to induce significant cytotoxicity in the HT-29 cells compared to fluphenazine alone (for concentrations <1 µM) or compared to sertraline alone at higher concentrations (>10 µM), demonstrating that the combination itself does not have improved anticancer activity compared to both drugs alone (Figure S11).

**Figure 11:**
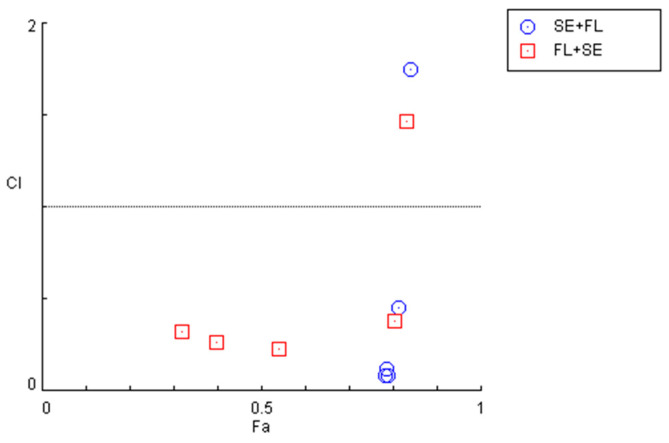
Graphical representation of combination index plot obtained from the CompuSyn Report for the combinations of sertraline IC_50_ + variable fluphenazine (blue) and fluphenazine IC_50_ + variable sertraline (red).

The quantification of the CI in these two combination regimens demonstrated that for concentrations under 10 µM, both combinations present synergism, with CI values under 1. Specifically, it was found that sertraline IC_50_ combined with 0.01, 0.1, 1, and 10 µM interact synergistically. The same occurs when fluphenazine IC_50_ is combined with 0.01, 0.1, 1, and 10 µM sertraline. These results are represented in Figure 11 and Table 7.

## 4. Discussion

Breast and colon cancer are among the most diagnosed types of cancers around the world, affecting both women and men [27]. Although surgery and chemotherapy play important roles in the therapy of these diseases, the development of drug resistance and the severe side effects that are associated with the chemotherapeutic agents significantly limit the efficacy of the treatments. The development of novel chemotherapeutic drugs is a process that involves elevated costs, an enormous amount of time, and several bureaucratic issues, that contribute to delaying the approval and arrival of these drugs to the market, and their application in clinical use. Therefore, the scientific community is focusing its research on the development of faster and more economical strategies, such as drug repurposing and drug combination.

Drug repurposing aims to find novel indications for drugs that are already approved in the market [28]. The use of these drugs is advantageous over the discovery of new drugs as repurposed drugs were already fully studied in previous clinical trials, resulting in available full data about their pharmacokinetics and pharmacodynamics profiles [29]. This means that only efficacy tests are necessary to confirm the pharmacological effect of the drug for other therapeutical indications, simplifying and accelerating the approval process of the drug. Drug combination refers to the use of cocktails with simultaneous administration of two or more drugs, aiming to enhance the efficacy of the treatment by using drugs with different mechanisms of action that can synergically act on different pathways involved in the tumorigenesis process [30].

In our previous work [22,23,24], we developed a new combination model consisting of antineoplastic and repurposed drugs to combat breast and colon cancer. We studied different classes of repurposed drugs, including antimalarial and CNS agents, alone and combined with several chemotherapeutic drugs, in two different cell lines (MCF-7 and HT-29). In a single treatment, we found pyronaridine and mefloquine to be the two most potent antimalarial drugs and sertraline and fluphenazine to be the two most cytotoxic CNS agents against both cell lines, demonstrating the potential of these repurposed drugs to be used as standalone agents in breast and colon cancer therapies. In combination regimens, we found that CNS drugs act synergistically with PTX and 5-FU in the reduction in MCF-7 and HT-29 cell viability, respectively, demonstrating the interesting profile of this drug class to be used as adjuvant agents to enhance the anticancer potential of antineoplastic drugs in these diseases.

Based on these findings, here we evaluated the combination of two other CNS drugs (edaravone and quetiapine) in combination with antineoplastic drugs in HT-29 and MCF-7 cancer cells. Edaravone is a pyrazolone derivative usually prescribed for the treatment of amyotrophic lateral sclerosis or after an acute cerebral infarction [31]. It was first approved in Japan and South Korea in 2015 and then in the US in 2017 [32,33]. Besides being a scavenger of oxygen free radicals, it also modulates several transcription factors, repressing NF-kB and activating Nrf2, acting as an oxidative stress modulator [34]. Nrf2 is a transcription factor that plays an important role in the response of cells to oxidative stress since it regulates the expression of multiple antioxidant key genes [35]. Under oxidative stress, this transcription factor translocates into the nucleus and modulates the expression of the antioxidant enzymes HO-1, thioredoxin-1, the peroxiredoxin gene family, and enzymes implanted in glutathione synthesis [36]. In cancer cells, Nrf2 is an important player in the development of drug resistance, impairing drug-mediated oxidative stress and contributing to cancer cell survival, by inhibiting apoptosis and promoting cell proliferation [7,37]. Nevertheless, some studies implicated this drug as a moderate anticancer agent [38,39,40,41]. A study demonstrated this drug has antiproliferative properties and is able to significantly enhance the anticancer and antimetastatic activities of irinotecan in a colon cancer model [38]. Other studies also demonstrated that this drug, used in combination with antineoplastic agents, can reduce the side effects associated with chemotherapy, such as renal dysfunction [42], neurotoxicity [43], and cardiotoxicity [44]. Quetiapine is an atypical antipsychotic drug that is used to treat schizophrenia and other neurologic disorders such as depression [45]. Recently, a study demonstrated that quetiapine is able to decrease the proliferation of brain cancer cells and induce glioblastoma-derived stem-like cell differentiation by inhibiting the Wnt/β-catenin signaling pathway [46]. Taking these data into account, we explored whether these drugs could possess anticancer activity properties against MCF-7 and HT-29 cancer cells and/or whether they could successfully enhance the anticancer effects of chemotherapy. First, cells were treated with increasing concentrations (0.01–100 µM) of each drug alone for 48 h to determine their IC_50_. We found that edaravone does not induce cytotoxic effects on the MCF-7 and HT-29 cancer cell lines, and, therefore, should not be used as a standalone agent for cancer therapy. On the other hand, quetiapine demonstrated significant anticancer activity against HT-29 colon cancer cells at concentrations of 50 and 100 µM, with an IC_50_ of about 15 µM. For the combined treatments, MCF-7 cells were treated with the IC_50_ values for DOX or PTX and combined with increasing concentrations (0.01–100 µM) of edaravone or quetiapine for 48 h. In the case of the HT-29 cells, the treatment consisted of the combination of 5-FU at IC_50_ with increasing concentrations (0.01–100 µM) of edaravone or quetiapine for 48 h. Here, it was found that the use of quetiapine synergistically enhances the anticancer activity of DOX for the management of breast cancer. Both CNS drugs significantly improved the cytotoxic potential of 5-FU in the HT-29 cells, with quetiapine synergistically interacting with the antineoplastic drug in this drug combination. Taken together, these results demonstrate that quetiapine can act as a chemosensitizer in combination therapies for breast and colon cancer. The mechanism of action behind these drug combinations should be further explored but studies suggest it may involve the Wnt/β-catenin signaling pathway [46].

Finally, we proposed a new model of combination consisting of the use of two repurposed drugs. To do so, we selected the most promising repurposed drugs from each drug class evaluated in our previous studies: pyronaridine and mefloquine (antimalarial drugs) and sertraline and fluphenazine (CNS drugs). MCF-7 and HT-29 cancer cell lines were treated with a combination of these drugs for 48 h, in a protocol consisting of the administration of a fixed concentration (IC_50_) of drug 1 with variable concentrations of drug 2. The results of this study demonstrate that the combination of antimalarial drugs is not advantageous over a single treatment, for either cell line, indicating that these repurposed drugs are more promising as standalone agents in breast and colon cancer therapies than in combination with other repurposed drugs. Nevertheless, our results support their use as chemosensitizers in combinations involving antineoplastic agents. Regarding the combination of CNS drugs, it was found that the combination of sertraline IC_50_ plus variable concentrations of fluphenazine synergistically reduced the viability of HT-29 cancer cells, with significant anticancer potential at lower concentrations (0.01, 1, and 10 µM) of fluphenazine. Interestingly, in the combination of the lowest concentration of 0.01 µM of fluphenazine with the IC_50_ value for sertraline (2.45 µM), which is also a very low concentration, it was possible to achieve more than 70% of HT-29 cell death, which demonstrates the promising anticancer profile of this drug combination for colon cancer therapy. Nevertheless, the aforementioned results must be further evaluated in other cell lines, especially in non-tumoral lines to evaluate their safety profile and confirm their selectivity for tumor cells. These drugs should also be evaluated in other cancer cell lines, such as prostate, lung, ovarian, and so on, to further explore their potential for other types of cancer. Even in breast and colon cancer, other cell lines such as resistant lines should be used to explore these combinations in other tumor subtypes. In the future, the mechanisms of the action behind these drugs in combination may also be further explored and their application in more complex models such as animals should also be evaluated.

Taken together, these results suggest that the CNS drug quetiapine can be used as an adjuvant agent in chemotherapy for breast and colon cancer. Furthermore, the combination of repurposed drugs, namely combining the CNS drugs sertraline and fluphenazine at lower doses, can also be further investigated for colon cancer novel therapies.

## Figures and Tables

**Table 1:** CI values and the respective fractional effect of different combinations of antineoplastic drugs plus CNS agents in MCF-7 cells. CI in bold indicates drug pairs that are synergic.

Combination (Drug A + Drug B)	Dose A	Dose B	Fractional Effect (Fa)	CI Value
DOX + edaravone	IC_50_	0.01	0.389	>10
		0.1	0.393	>10
		1	0.343	>10
		10	0.381	>10
		50	0.370	>10
		100	0.376	>10
PTX + edaravone	IC_50_	0.01	0.255	>10
		0.1	0.307	>10
		1	0.240	>10
		10	0.247	>10
		50	0.240	>10
		100	0.274	>10
DOX + quetiapine	IC_50_	0.01	0.399	**0.528**
		0.1	0.464	**0.363**
		1	0.451	**0.390**
		10	0.530	**0.252**
		50	0.554	**0.231**
		100	0.562	**0.232**
PTX + quetiapine	IC_50_	0.01	0.173	**0.182**
		0.1	0.264	**0.049**
		1	0.283	**0.043**
		10	0.231	**0.186**
		50	0.287	**0.295**
		100	0.567	**0.027**

**Table 2:** CI values and the respective fractional effect of different combinations of 5-FU plus CNS agents in HT-29 cells. CI in bold indicates concentrations of drug pairs that are synergic.

Combination (Drug A + Drug B)	Dose A	Dose B	Fractional Effect (Fa)	CI Value
5-FU + edaravone	IC_50_	0.01	0.261	>10
		0.1	0.406	>10
		1	0.367	>10
		10	0.529	>10
		50	0.458	>10
		100	0.645	>10
5-FU + quetiapine	IC_50_	0.01	0.307	**0.095**
		0.1	0.398	**0.072**
		1	0.374	**0.102**
		10	0.654	**0.036**
		50	0.643	**0.061**
		100	0.775	**0.026**

**Table 3:** IC_50_ values of antineoplastic and repurposed drugs in MCF-7 breast and HT-29 colon cancer cells. Only tested drugs with IC_50_ values under 50 µM are displayed. Bold indicates repurposed drugs selected for this study regarding the evaluation of this novel model of combination consisting of repurposed drugs. Adapted from [22,23,24].

	Drug	HT-29	MCF-7
		IC_50_ (µM)	IC_50_ (µM)
CNS	**Fluphenazine**	**1.86**	**2.68**
	Fluoxetine	6.12	7.78
	Benztropine	18.23	21.71
	Thioridazine	4.26	5.72
	**Sertraline**	**2.45**	**2.22**
	Entacapone	40.89	ND
	Tolcapone	35.47	ND
	Edaravone	>50	>50
	Quetiapine	15.19	>50
Antimalarials	Artesunate	17.88	11.60
	Chloroquine	32.13	N.D.
	**Mefloquine**	**2.18**	**1.24**
	Cycloguanil	N.D.	20.30
	Piperazine	N.D.	3.24
	Primaquine	N.D.	29.90
	**Pyronaridine**	**2.43**	**1.39**
	Tafenoquine	N.D.	2.60

**Table 4:** CI values and the respective fractional effect of drug combinations using antimalarial drugs in MCF-7 cells. CI in bold indicates concentrations of drug pairs that are synergic.

Combination (Drug A + Drug B)	Dose A	Dose B	Fractional Effect (Fa)	CI Value
Mefloquine + Pyronaridine	IC_50_	0.01	0.204	>10
		0.1	0.242	7.063
		1	0.339	1.537
		10	0.582	**0.205**
		50	0.623	**0.664**
		100	0.618	1.324
Pyronaridine + Mefloquine	IC_50_	0.01	0.538	**0.025**
		0.1	0.514	**0.038**
		1	0.547	**0.096**
		10	0.621	**0.279**
		50	0.619	1.358
		100	0.632	2.272

**Table 5:** CI values and the respective fractional effect of drug combinations using CNS drugs in MCF-7 cells. CI in bold indicates concentrations of drug pairs that are synergic.

Combination (Drug A + Drug B)	Dose A	Dose B	Fractional Effect (Fa)	CI Value
Sertraline + Fluphenazine	IC_50_	0.01	0.192	8.434
		0.1	0.177	>10
		1	0.255	5.102
		10	0.601	**0.351**
		50	0.618	1.269
		100	0.629	2.197
Fluphenazine + Sertraline	IC_50_	0.01	0.156	>10
		0.1	0.196	>10
		1	0.218	>10
		10	0.619	**0.250**
		50	0.621	**0.967**
		100	0.630	1.676

**Table 6:** CI values and the respective fractional effect of drug combinations using antimalarial drugs in HT-29 cells. CI in bold indicates concentrations of drug pairs that are synergic.

Combination (Drug A + Drug B)	Dose A	Dose B	Fractional Effect (Fa)	CI Value
Mefloquine + Pyronaridine	IC_50_	0.01	0.891	**0.164**
		0.1	0.889	**0.169**
		1	0.891	**0.192**
		10	0.893	**0.439**
		50	0.904	1.426
		100	0.907	2.643
Pyronaridine + Mefloquine	IC_50_	0.01	0.385	**0.373**
		0.1	0.219	**0.654**
		1	0.600	**0.263**
		10	0.850	**0.256**
		50	0.875	**0.777**
		100	0.877	1.460

**Table 7:** CI values and the respective fractional effect of drug combinations using CNS drugs in HT-29 cells. CI in bold indicates concentrations of drug pairs that are synergic.

Combination (Drug A + Drug B)	Dose A	Dose B	Fractional Effect (Fa)	CI Value
Sertraline + Fluphenazine	IC_50_	0.01	0.784	**0.081**
		0.1	0.789	**0.083**
		1	0.786	**0.122**
		10	0.815	**0.451**
		50	0.840	1.749
		100	0.838	3.464
Fluphenazine + Sertraline	IC_50_	0.01	0.319	**0.322**
		0.1	0.399	**0.262**
		1	0.542	**0.230**
		10	0.805	**0.380**
		50	0.831	1.466
		100	0.836	2.814

## Data Availability

Not applicable.

## Supplementary Materials

The following supporting information can be downloaded at: https://www.mdpi.com/article/10.3390/cimb44100335/s1.
