# Supplementary material for: Novel Strategies for Cancer Combat: Drug Combination Using Repurposed Drugs Induces Synergistic Growth Inhibition of MCF-7 Breast and HT-29 Colon Cancer Cells
Source: Curr Issues Mol Biol. 2022 Oct 16;44(10):4930–49. doi: 10.3390/cimb44100335 (PMC9601176; doi:10.3390/cimb44100335)
Supplement: Supplementary file 1 [file cimb-44-00335-s001.zip › cimb-1960090-supplementary.pdf]

Novel Strategies for Cancer Combat: Drug Combination using Repurposed Drugs Induces Synergistic Growth Inhibition of MCF-7 Breast and HT-29 Colon Cancer Cells

Diana Duarte [1,2,3], Inês Guerreiro [1] and Nuno Vale [1,3,4,*]

1 OncoPharma Research Group, Center for Health Technology and Services Research (CINTESIS), Rua Dr. Plácido da Costa, s/n, 4200-450 Porto, Portugal
2 Faculty of Pharmacy, University of Porto, Rua Jorge Viterbo Ferreira, 228, 4050-313 Porto, Portugal
3 Department of Community Medicine, Health Information and Decision (MEDCIDS), Faculty of Medicine, University of Porto, Rua Dr. Plácido da Costa, s/n, 4200-450 Porto, Portugal
4 CINTESIS@RISE, Faculty of Medicine, University of Porto, Al. Prof. Hernâni Monteiro, 4200-319 Porto, Portugal

* Correspondence: nunovale@med.up.pt; Tel.: +351-220426537

Supplementary Material

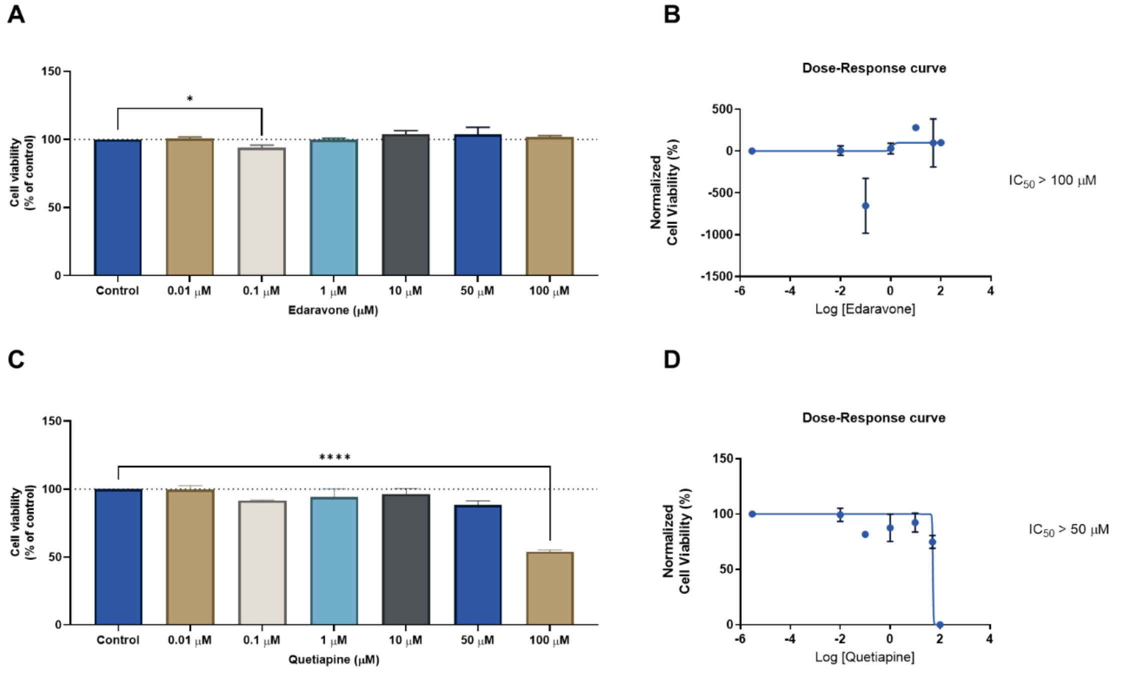

Figure S1. Cellular viability results of MCF-7 cells after treatment with the CNS drugs edaravone (top) and quetiapine (bottom). (A) MTT results and (B) dose-response curve of MCF-7 cells treated with edaravone. (C) MTT results and (D) dose-response curve of MCF-7 cells treated with quetiapine. Cells were incubated with increasing concentrations of each drug for 48 h and cell viability was evaluated using an MTT assay. Dose-response curves were obtained by normalization of cell viability results against the logarithm of the concentration of each drug. The IC_{50} value was determined using GraphPad. Experiments were performed three times independently (n = 3). * and **** indicate significative results at $p < 0.05$ and $p < 0.0001$, respectively.

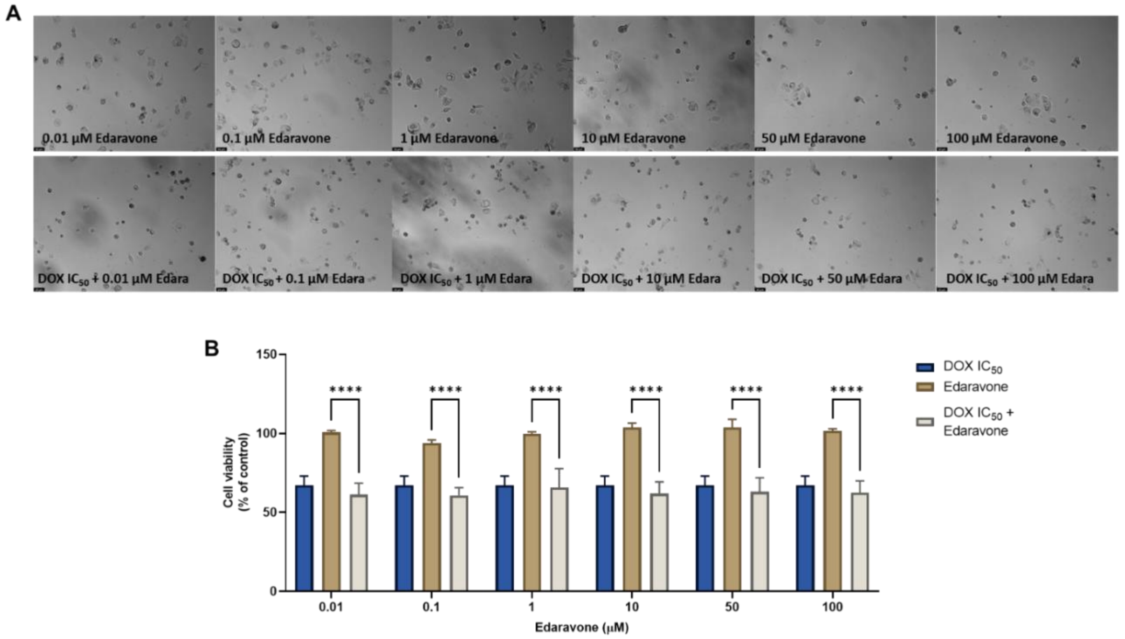

Figure S2. Morphological analysis (A) and cellular viability (B) results of MCF-7 cells after a single and combined treatment of DOX and edaravone. Cells were incubated with increasing concentrations of edaravone, alone and combined with a fixed concentration (IC_{50}) of DOX for 48 h. Cell viability was evaluated using an MTT assay. Experiments were performed three times independently ($n = 3$). **** indicate significative results at $p < 0.0001$.

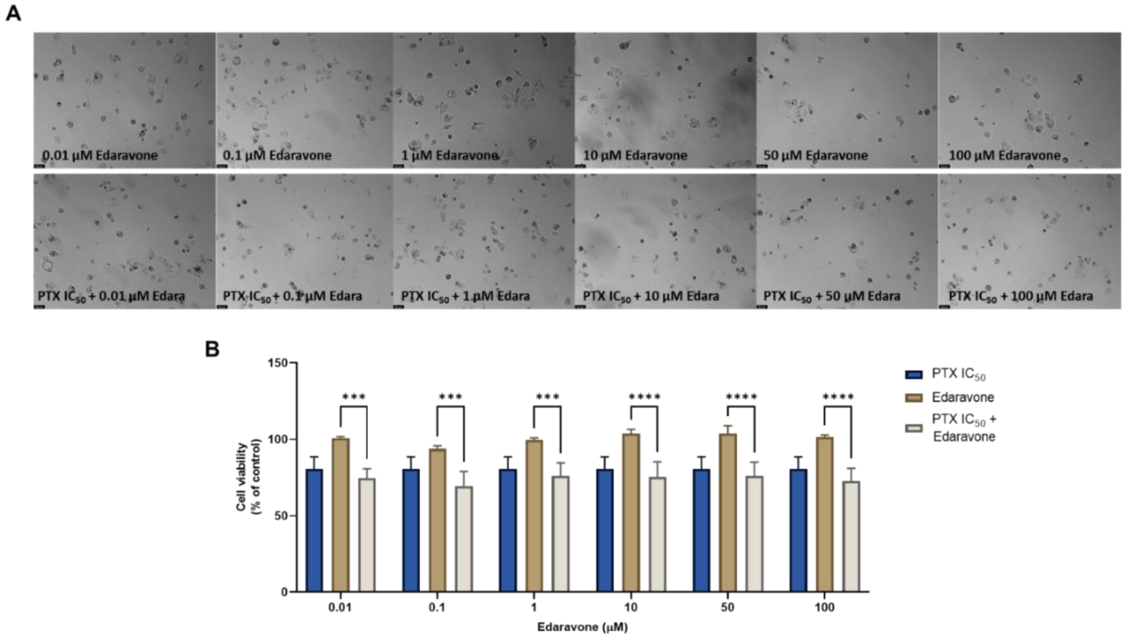

Figure S3. Morphological analysis (A) and cellular viability (B) results of MCF-7 cells after a single and combined treatment of PTX and edaravone. Cells were incubated with increasing concentrations of edaravone, alone and combined with a fixed concentration (IC_{50}) of PTX for 48 h. Cell viability was evaluated using an MTT assay. Experiments were performed three times independently ($n = 3$). *** and **** indicate significative results at $p < 0.001$ and $p < 0.0001$, respectively.

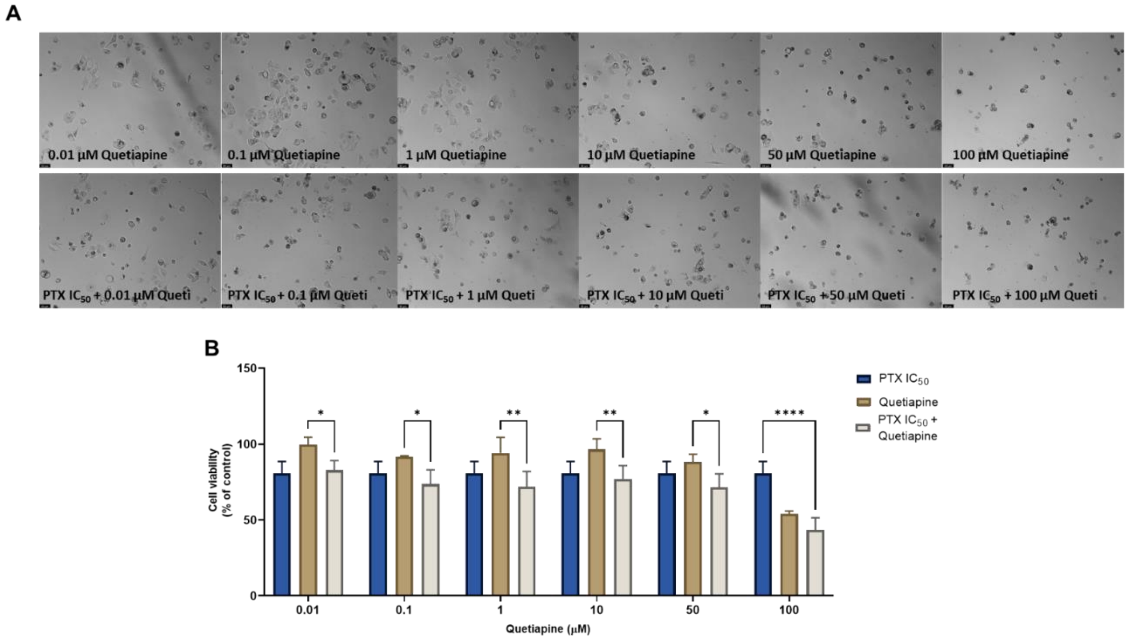

Figure S4. Morphological analysis (A) and cellular viability (B) results of MCF-7 cells after a single and combined treatment of PTX and quetiapine. Cells were incubated with increasing concentrations of quetiapine, alone and combined with a fixed concentration (IC_{50}) of PTX for 48 h. Cell viability was evaluated using an MTT assay. Experiments were performed three times independently (n = 3). *, ** and **** indicate significative results at $p < 0.05$, $p < 0.01$ and $p < 0.0001$, respectively.

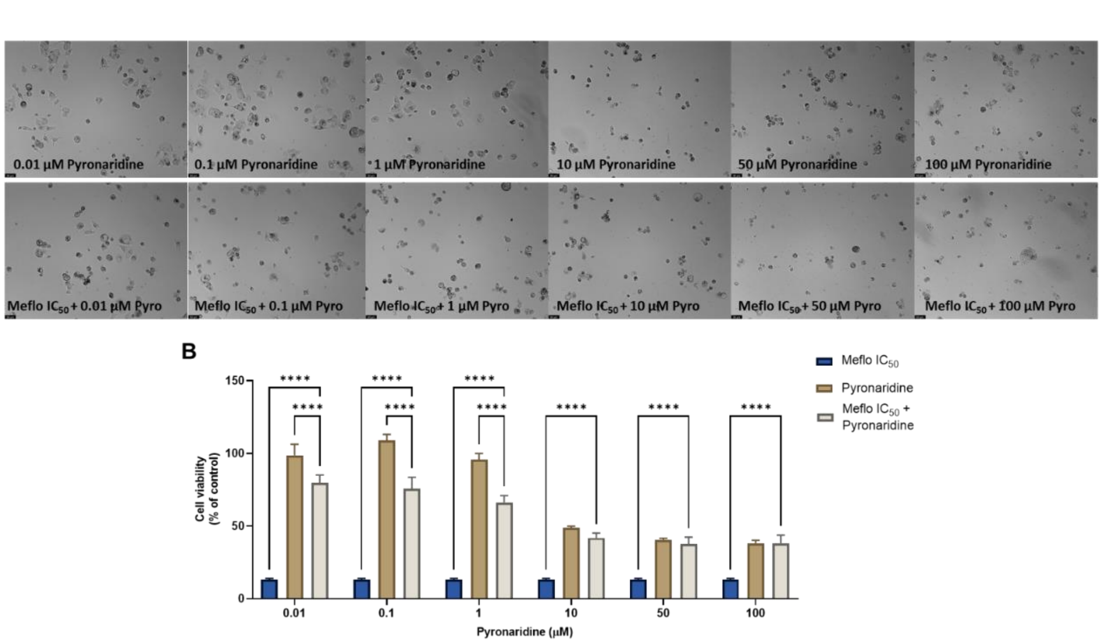

Figure S5. Morphological analysis (A) and cellular viability (B) results of MCF-7 cells after a single and combined treatment of mefloquine and pyronaridine. Cells were incubated with increasing concentrations of pyronaridine, alone and combined with a fixed concentration (IC_{50}) of mefloquine for 48 h. Cell viability was evaluated using an MTT assay. Experiments were performed three times independently (n = 3). **** indicate significative results at $p < 0.0001$.

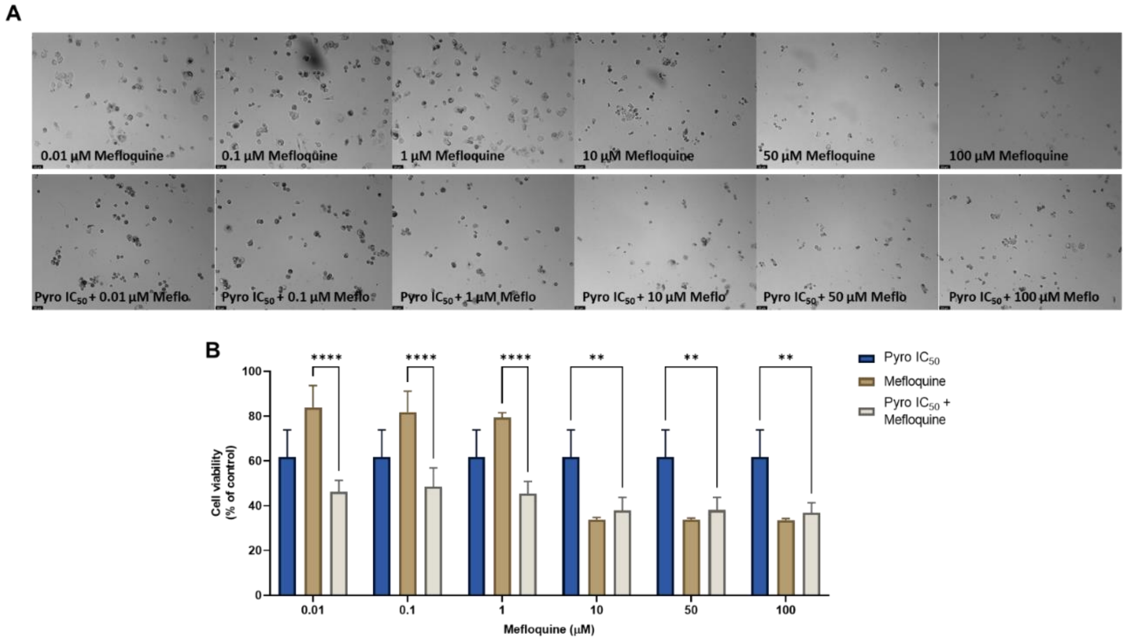

Figure S6. Morphological analysis (A) and cellular viability (B) results of MCF-7 cells after a single and combined treatment of pyronaridine and mefloquine. Cells were incubated with increasing concentrations of mefloquine, alone and combined with a fixed concentration (IC_{50}) of pyronaridine for 48 h. Cell viability was evaluated using an MTT assay. Experiments were performed three times independently ($n = 3$). ** and **** indicate significative results at $p < 0.01$ and $p < 0.0001$, respectively.

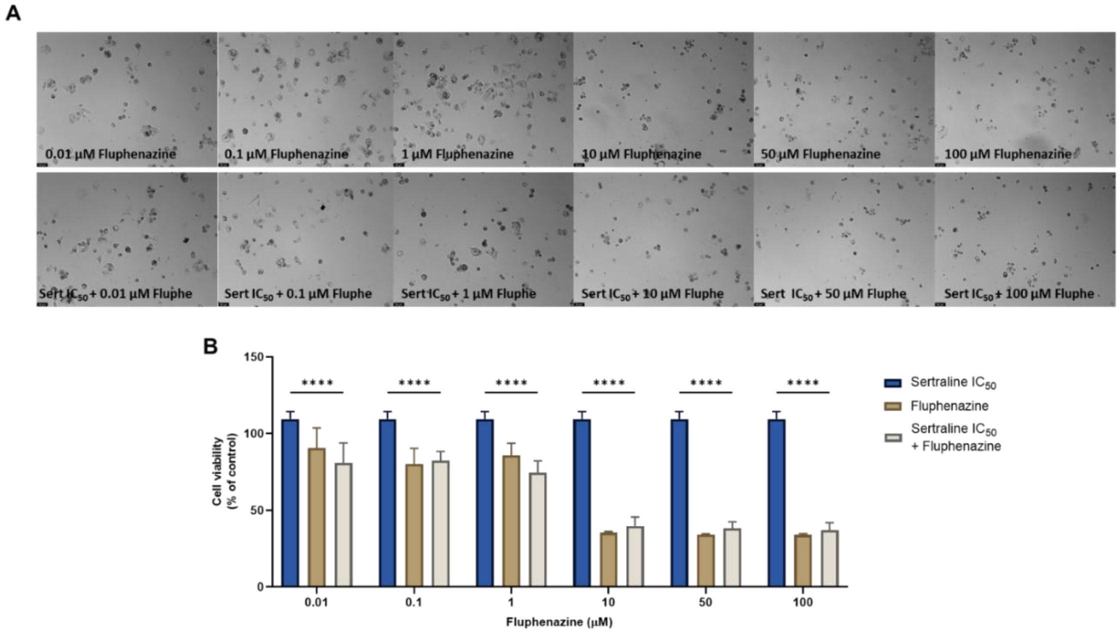

Figure S7. Morphological analysis (A) and cellular viability (B) results of MCF-7 cells after a single and combined treatment of fluphenazine and sertraline. Cells were incubated with increasing concentrations of fluphenazine, alone and combined with a fixed concentration (IC_{50}) of sertraline for 48 h. Cell viability was evaluated using an MTT assay. Experiments were performed three times independently ($n = 3$). **** indicate significative results at $p < 0.0001$.

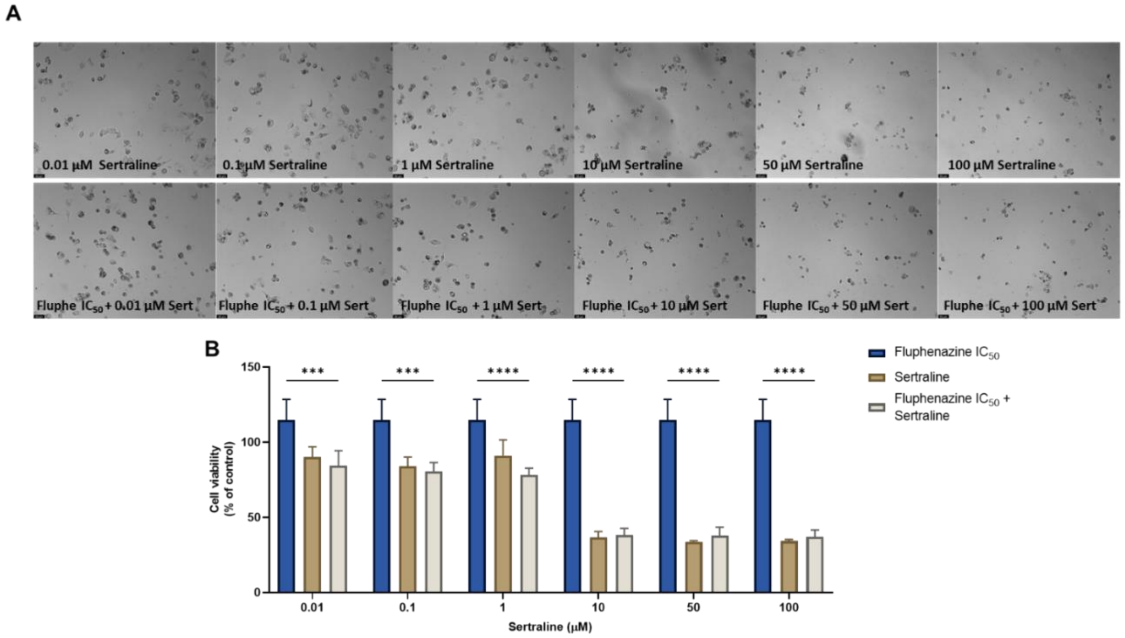

Figure S8. Morphological analysis (A) and cellular viability (B) results of MCF-7 cells after a single and combined treatment of sertraline and fluphenazine. Cells were incubated with increasing concentrations of sertraline, alone and combined with a fixed concentration (IC_{50}) of fluphenazine for 48 h. Cell viability was evaluated using an MTT assay. Experiments were performed three times independently ($n = 3$). *** and **** indicate significative results at $p < 0.001$ and $p < 0.0001$, respectively.

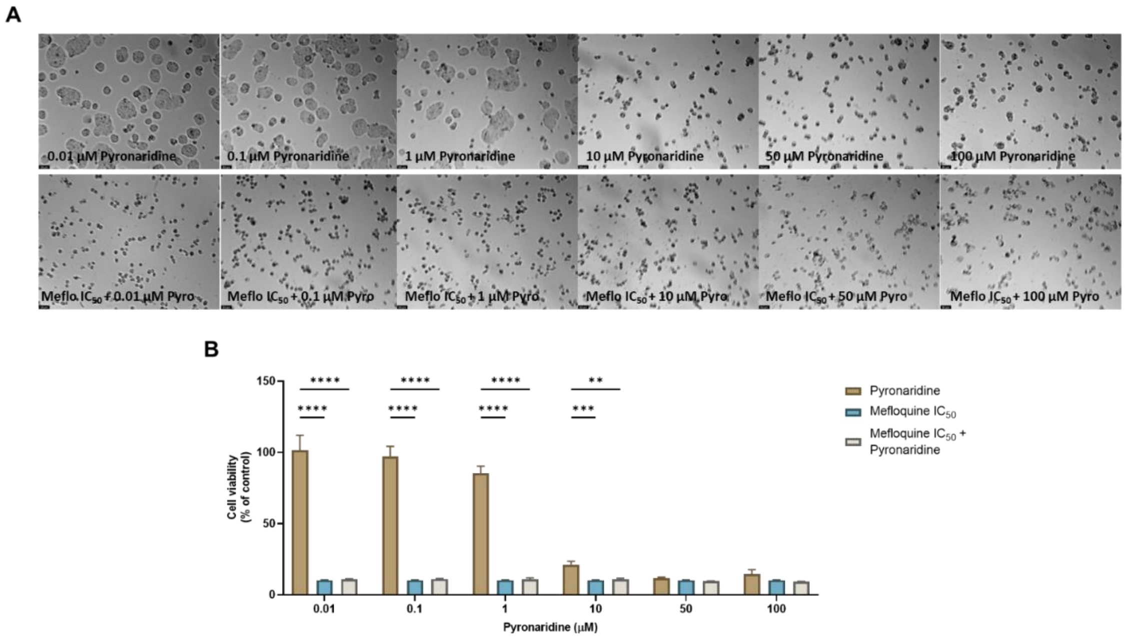

Figure S9. Morphological analysis (A) and cellular viability (B) results of HT-29 cells after a single and combined treatment of mefloquine and pyronaridine. Cells were incubated with increasing concentrations of pyronaridine, alone and combined with a fixed concentration (IC_{50}) of mefloquine for 48 h. Cell viability was evaluated using an MTT assay. Experiments were performed three times independently ($n = 3$). **, *** and **** indicate significative results at $p < 0.01$, $p < 0.001$ and $p < 0.0001$, respectively.

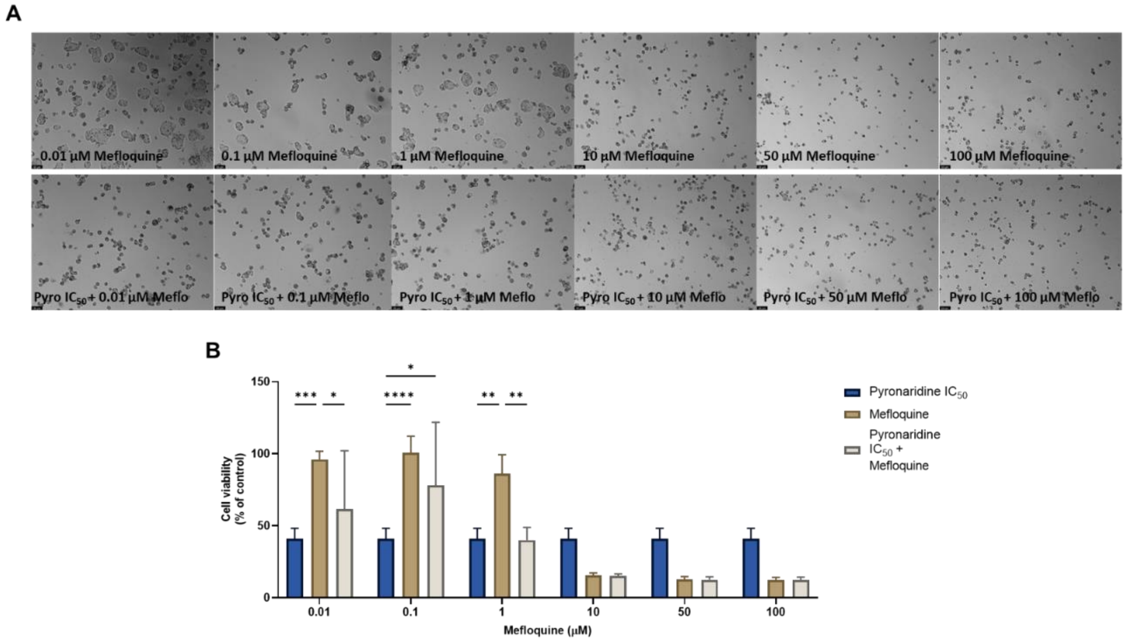

Figure S10. Morphological analysis (A) and cellular viability (B) results of HT-29 cells after a single and combined treatment of mefloquine and pyronaridine. Cells were incubated with increasing concentrations of mefloquine, alone and combined with a fixed concentration (IC_{50}) of pyronaridine for 48 h. Cell viability was evaluated using an MTT assay. Experiments were performed three times independently ($n = 3$). *, **, *** and **** indicate significative results at $p < 0.05$, $p < 0.01$, $p < 0.001$ and $p < 0.0001$, respectively.

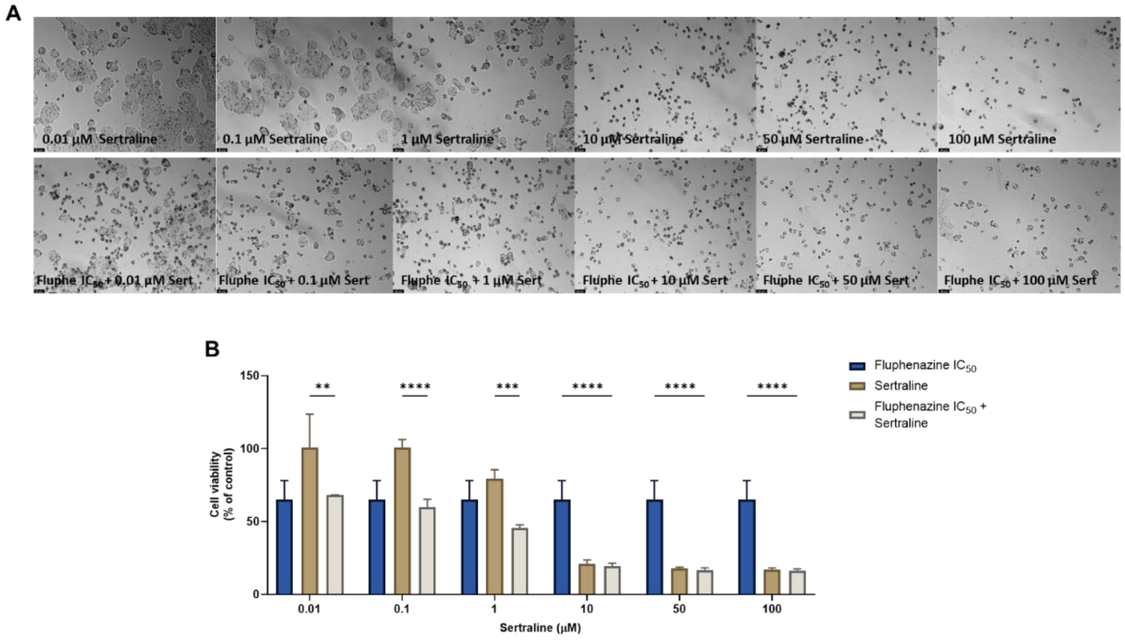

Figure S11. Morphological analysis (A) and cellular viability (B) results of HT-29 cells after a single and combined treatment of fluphenazine and sertraline. Cells were incubated with increasing concentrations of sertraline, alone and combined with a fixed concentration (IC_{50}) of fluphenazine for 48 h. Cell viability was evaluated using an MTT assay. Experiments were performed three times independently ($n = 3$). **, *** and **** indicate significative results at $p < 0.01$, $p < 0.001$ and $p < 0.0001$, respectively.
